# Elevation of C-reactive protein levels in patients with transfusion-related acute lung injury

**DOI:** 10.18632/oncotarget.12872

**Published:** 2016-10-25

**Authors:** Rick Kapur, Michael Kim, Matthew T. Rondina, Leendert Porcelijn, John W. Semple

**Affiliations:** ^1^ Keenan Research Centre for Biomedical Science, St. Michael's Hospital, Toronto, ON, Canada; ^2^ The Toronto Platelet Immunobiology Group, St. Michael's Hospital, Toronto, ON, Canada; ^3^ Canadian Blood Services, Toronto, ON, Canada; ^4^ Molecular Medicine Program, University of Utah, Salt Lake City, UT, United States; ^5^ Department of Internal Medicine, University of Utah, Salt Lake City, UT, United States; ^6^ Department of Internal Medicine, George E. Wahlen Salt Lake City VAMC, University of Utah, Salt Lake City, UT, United States; ^7^ Department of Thrombocyte and Leukocyte Serology, Sanquin Diagnostic Services, Amsterdam, The Netherlands; ^8^ Department of Pharmacology, Medicine, and Laboratory Medicine and Pathobiology, University of Toronto, Toronto, ON, Canada

**Keywords:** CRP, TRALI, human TRALI, TRALI risk factor, TRALI first hit

## Abstract

Transfusion-related acute lung injury (TRALI) is the leading cause of transfusion-related fatalities and is characterized by the onset of acute respiratory distress within six hours following blood transfusion. In most cases, donor antibodies are suggested to be involved, however, the pathogenesis is poorly understood. A two-hit model is generally assumed to underlie TRALI pathogenesis where the first hit consists of a patient predisposing factor such as inflammation and the second hit is due to donor antibodies present in the transfused blood. We recently demonstrated that the acute phase protein C-reactive protein (CRP) could enhance murine anti-major histocompatibility complex (MHC) class I-mediated TRALI. Whether CRP is increased in human TRALI patients which would support its role as a risk factor for human TRALI, is currently unknown. For that purpose, we measured CRP levels in the plasma of human TRALI patients and found CRP levels to be significantly elevated compared to transfused control patients. These data support the notion that CRP may be a novel first hit risk factor in human TRALI and that modulation of CRP levels could be an effective therapeutic strategy for this serious adverse event of transfusion.

## INTRODUCTION

Transfusion-related acute lung injury (TRALI) is the leading cause of transfusion-related mortality and is characterized by acute respiratory distress within six hours following blood transfusions [1, 2]. Apart from supportive measures such as oxygen and ventilation, no therapy is currently available for TRALI. Most TRALI cases have been associated to the presence of human leucocyte antigen (HLA)- or human neutrophil antigen (HNA) antibodies in the donor blood [3, 4]. A two-hit model has been proposed for antibody-mediated TRALI where the first hit comprises patient predisposing factors such as inflammation and the second hit is due to donor antibodies in the transfused blood [1]. Other first-hit risk factors for TRALI include chronic alcohol abuse, liver surgery, smoking, shock, higher peak airway pressure while undergoing mechanical ventilation, and positive intravascular fluid balance [5]. More specifically, systemic inflammation characterized by elevated recipient interleukin (IL)-6 [6] and IL-8 levels [5-7] also appears to be a major risk factor for TRALI induction. In contrast, inflammatory conditions like sepsis can also trigger acute lung injury (ALI) without any transfusions. Although both TRALI and ALI appear to be similar with regard to the occurence of acute lung injury and respiratory distress, their underlying pathogenic mechanisms are different.

We recently demonstrated that C-reactive protein (CRP), an acute phase protein widely used as a clinical biomarker of infections and inflammation, enhances anti-major histocompatibility complex (MHC) class I-mediated TRALI in BALB/c mice [8]. Mechanistically, mice were resistant to TRALI induction upon injection of anti-MHC class I antibody or CRP alone, however, when CRP was infused together with the antibody, a synergistic increase was observed in the levels of the PMN chemoattractant macrophage inflammatory protein-2 (MIP-2, murine homologue of human IL-8) [8]. This was accompanied by a synergistic increase in pulmonary PMN accumulation resulting in increased signs of acute lung injury as demonstrated by assessment of pulmonary edema and lung histology [8].

In light of these findings in mice, we assessed whether CRP levels are indeed increased in human TRALI patients which is an essential step to establish if CRP may be a risk factor for TRALI induction and if modulation of CRP levels in humans could be a therapeutic approach to combat TRALI.

## RESULTS

**Figure 1:**
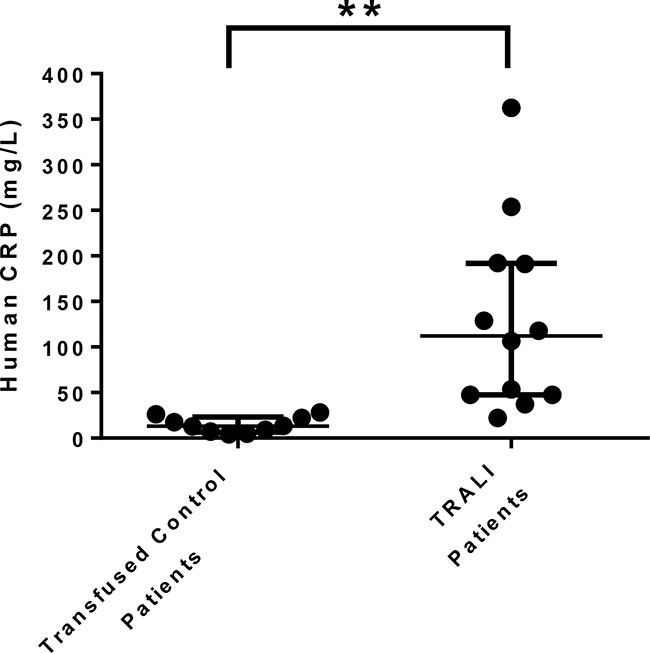
CRP levels are increased in human TRALI patients CRP levels were measured in plasma from transfused control patients (who did not undergo any adverse pulmonary transfusion reactions including TRALI, n=10) and from TRALI patients (n=12). A one-sided and unpaired t-test was performed, **: P<0.01. Error bars represent median with interquartile range.

CRP levels were measured in plasma samples from human TRALI patients (n=12) and in control orthopedic surgery patients that had not been hospitalized nor had infections within one month prior to surgery and received allogeneic blood transfusions post-operatively without any pulmonary transfusion reactions (n=10). TRALI samples were collected for CRP analysis within 24-48 hours upon transfusion and for the control group blood samples were collected within 24 hours following transfusion (which occurred on post-operative days 3-4). The patient characteristics and clinical features are listed in Table 1. CRP levels were found to be significantly increased in TRALI patients compared to transfused control patients, with a median (interquartile range) of 112.2 mg/L (47.45-192) versus 13.02 mg/L (6.48-23.01), respectively (Figure 1). The mean values were 130 mg/L (+/− 103.3 mg/L SD) for TRALI patients versus 14.51 mg/L (+/− 8.7 mg/L SD) for transfused control patients.

**Table 1:** Human patient characteristics

Patient sample	Gender	Age (years)	Type of Transfusion	Clinical condition	Additional clinical information	Additional laboratory testing
1	F	62	Non-autologous packed RBCs	Orthopedic surgery; elective, unilateral TKA. Sample collection post-transfusion.	Diabetes	N/A
2	M	64	Non-autologous packed RBCs	Orthopedic surgery; elective, unilateral THA. Sample collection post-transfusion.	Diabetes	N/A
3	F	70	Non-autologous packed RBCs	Orthopedic surgery; elective, unilateral THA. Sample collection post-transfusion.	N/A	N/A
4	M	67	Non-autologous packed RBCs	Orthopedic surgery; elective, unilateral THA. Sample collection post-transfusion.	N/A	N/A
5	F	76	Non-autologous packed RBCs	Orthopedic surgery; elective, unilateral TKA. Sample collection post-transfusion.	Diabetes	N/A
6	F	80	Non-autologous packed RBCs	Orthopedic surgery; elective, unilateral TKA. Sample collection post-transfusion.	Cardio-vascular disease	N/A
7	M	62	Non-autologous packed RBCs	Orthopedic surgery; elective, unilateral TKA. Sample collection post-transfusion.	Diabetes	N/A
8	F	58	Non-autologous packed RBCs	Orthopedic surgery; elective, unilateral THA. Sample collection post-transfusion.	N/A	N/A
9	F	69	Non-autologous packed RBCs	Orthopedic surgery; elective, unilateral TKA. Sample collection post-transfusion.	N/A	N/A
10	M	78	Non-autologous packed RBCs	Orthopedic surgery; elective, unilateral TKA. Sample collection post-transfusion.	N/A	N/A
11	F	40	Whole blood buffy coat, packed RBCs	TRALI	Lymphoma with suspected hemorrhaging post-biopsy	Donor leukocyte antibodies detected (anti-HLA A23, DQ2, DQ5, but no matching cognate antigen on patient leukocytes)
12	F	29	Packed RBCs, apheresis platelets	TRALI	Post-partum hemorrhaging	Donor leukocyte antibodies detected (anti-HNA1a, with matching cognate antigen on patient leukocytes)
13	M	63	Apheresis platelets	TRALI	Aplastic anemia	Donor leukocyte antibodies detected (anti-HLA Cw5, but no matching cognate antigen on patient leukocytes)
14	M	69	Packed RBCs	TRALI	Anemia, chronic kidney disease	Donor leukocyte antibodies detected (anti-HLA-DR4, with matching cognate antigen on patient leukocytes)
15	F	39	Packed RBCs	TRALI	Anemia after chemo-therapy (breast cancer). Kearns–Sayre syndrome.	Donor leukocyte antibodies detected (anti-HLA, with matching cognate antigen on patient leukocytes)
16	M	48	Packed RBCs	TRALI	Solid tumor surgery.	Donor leukocyte antibodies detected (anti-IgM HLA-A1, but no matching cognate antigen on patient leukocytes)
17	F	33	Packed RBCs, fresh frozen plasma	TRALI	Post-cesarean section hemorrhagic shock	No donor leukocyte antibodies detected
18	M	67	Packed RBCs	TRALI	Leukemia, chronic obstructive pulmonary disease, active lung cancer	No donor leukocyte antibodies detected
19	F	65	Packed RBCs	TRALI	Transfused for cardiac support (history of percutaneous transluminal coronary angioplasty). Stable pancreatitis.	No donor leukocyte antibodies detected
20	M	24	Packed RBCs	TRALI	Anemia and oliguria post-laparotomy	No donor leukocyte antibodies detected
21	M	53	Fresh frozen plasma	TRALI	Anemia due to gastro-intestinal bleeding	No donor leukocyte antibodies detected
22	F	40	Packed RBCs, whole blood buffy coat, fresh frozen plasma	TRALI	Anemia due to hemorrhaging following gastric surgery	Donor leukocyte antibodies detected (cytotoxic antibodies reactive with patient leukocytes)

Human plasma samples from transfused control patients (which did not undergo any TRALI or pulmonary reactions, patient samples 1-10), and human TRALI plasma samples (Patient samples 11-22), M: male. F: female. N/A: not applicable. TKA: total knee arthroplasty. THA: total hip arthroplasty.

## DISCUSSION

The finding that CRP levels are significantly elevated in human TRALI patients (Figure 1) suggests that CRP may indeed be a risk factor and first hit for TRALI induction. The plasma half-life of CRP is approximately 19 hours [9] and the high concentrations of CRP in the plasma of TRALI patients (within 24-48 hrs post-transfusion, Figure 1) compared with controls suggests that a high-degree of inflammation is present. This first hit of inflammation signified by the increased levels of CRP together with the second hit conveyed by the specific antibodies present in the transfused blood product may be a key factor enabling TRALI reactions. This was illustrated in a murine model where it was shown that CRP together with anti-MHC class I antibodies synergistically enhanced MIP-2 levels (murine homologue of IL-8, a known risk factor for human TRALI [5, 7]) and pulmonary neutrophil accumulation leading to acute lung injury [8]. Notably, CRP or the anti-MHC class I antibody alone were insufficient to trigger a TRALI reaction [8]. Additional mechanisms of action could be that CRP may enhance the PMN respiratory burst as was demonstrated previously in a setting of thrombocytopenia [10] and reactive oxygen species have been suggested to be critical in TRALI induction [11-13]. Additionally, anti-MHC class I antibodies and CRP may synergize in targeting the pulmonary endothelium as both the antibody as well as CRP can directly bind to and affect endothelial cells [11, 14]. In addition, CRP has been shown to be involved in the activation of the complement cascade via C1q [15] so perhaps CRP may also affect the regulation of C5a which has been suggested to be involved in inducing murine TRALI [11].

CRP is member of the pentraxin family and is composed of 5 identical, nonglycosylated 206 amino acids protomers which together form a noncovalently linked annular symmetrical pentameric molecule [9]. Pentameric CRP is the native form of CRP which circulates in plasma and is upregulated during acute infection and inflammation [9] although some studies have observed the presence of monomeric CRP [16-18]. It is, however, unlikely that monomeric CRP participates in TRALI as monomeric CRP has been linked to different types of disease pathologies like atherosclerosis and myocardial infarction without antibody involvement [16-18]. In contrast, thrombocytopenia usually occurs in TRALI [19-22] and antibodies are frequently involved [1]. Moreover, we have shown that infusion of pentameric CRP in conjunction with anti-MHC class I antibodies synergistically enhanced TRALI reactions leading to thrombocytopenia [8]. Further research is, however, required to fully establish a monomeric contribution of CRP in TRALI.

Platelets are classically known for their hemostatic function but have been suggested to play a pathogenic role in TRALI [23, 24]. It is now recognized they have a potent ability to elicit multiple immune functions [25, 26] including the secretion of extracellular platelet microparticles which contain, for example, respiratory competent mitochondria [27]. Interestingly, high levels of extracellular mitochondria were recently identified in platelet transfusion concentrates which caused adverse acute transfusion reactions [27]. Perhaps these extracellular mitochondria enhance inflammation in the TRALI patient through induction of CRP, however, additional studies will be required to establish the actual presence of such a mechanism.

In summary, our data indicate that CRP levels are significantly upregulated in the plasma of TRALI patients and thus monitoring CRP levels prior to transfusion may be useful and down-regulating CRP levels could be a novel therapeutic approach to explore in TRALI.

## MATERIALS AND METHODS

### Study approval

Human TRALI samples were diagnosed and obtained with approval from Sanquin Diagnostic Services, Amsterdam, The Netherlands (Dr. L. Porcelijn). All TRALI samples were obtained and collected strictly under the following conditions (as ascertained by transfusion medicine specialists): 1) Newly developing acute respiratory distress: PaO2/FiO2 ratio (ratio of arterial oxygen partial pressure to fractional inspired oxygen) < 300 mmHg or arterial oxygen saturation < 90% at room air; 2) Newly developed or worsened bilateral pulmonary infiltrates indicative of pulmonary edema on chest X-ray; 3) Emergence of all symptoms within 6 hours upon blood transfusion; 4) Exclusion of cardiac ischemia and transfusion associated circulatory overload (TACO).

If patients had major risk factors for acute lung injury, such as pneumonia, sepsis, aspiration, multiple fractures or pancreatitis, they were excluded from this study except if the patient was stable and the risk factor did not appear to cause acute lung injury and the onset of acute lung injury was triggered by the blood transfusion.

Human transfused control samples were obtained from Dr. M. T. Rondina (University of Utah; Intermountain Health Care Institutional Review Boards approved this study in which samples were collected prospectively). Patient characteristics and clinical features for both TRALI and transfused control samples are described in Table 1. All samples were obtained with informed consent from the patients in accordance with the Declaration of Helsinki.

### CRP measurements

TRALI samples were collected for CRP analysis within 24-48 hours upon transfusion and for the control group blood samples were collected within 24 hours following transfusion, which occurred 3-4 days after the orthopedic surgery. CRP was measured from plasma using a standardized immunoturbidimetric assay, in which the human CRP from the samples agglutinates when incubated with latex particles coated with polyclonal rabbit anti-human CRP antibodies (Denka Seiken Co., ltd, Niigata, Japan). The aggregrates were subsequently determined turbidometrically using the Beckman AU680, Beckman Coulter, USA.
